# Low-grade chronic inflammation is attenuated by exercise training in obese adults through down-regulation of *ASC* gene in peripheral blood: a pilot study

**DOI:** 10.1186/s12263-020-00674-0

**Published:** 2020-08-27

**Authors:** Elisa Barrón-Cabrera, Karina González-Becerra, Gustavo Rosales-Chávez, Alondra Mora-Jiménez, Iván Hernández-Cañaveral, Erika Martínez-López

**Affiliations:** 1 Institute of Translational Nutrigenetics and Nutrigenomics, Department of Molecular Biology and Genomics, Health Sciences University Center, University of Guadalajara, Sierra Mojada 950, zip code, 44340 Guadalajara, Jalisco México; 2 Respiratory Therapy Unit, Health Sciences University Center, University of Guadalajara, Sierra Mojada 950, zip code, 44340 Guadalajara, Jalisco México; 3 Microbiology and Pathology Department, Department of Molecular Biology and Genomics, Health Sciences University Center, University of Guadalajara, Sierra Mojada 950, zip code, 44340 Guadalajara, Jalisco México

**Keywords:** Nutrition, Diet, Exercise, Inflammation

## Abstract

**Background:**

Obesity is characterized by low-grade chronic inflammation and an excess of adipose tissue. The *ASC* gene encodes a protein that is part of the NLRP3 inflammasome, a cytosolic multiprotein complex that is associated with inflammation and metabolic alterations. To our knowledge, there is no evidence regarding *ASC* gene activity in obese adults in response to lifestyle modifications.

**Purpose:**

To evaluate the effect of hypocaloric diet and moderate-intensity structured exercise intervention on *ASC* gene expression and inflammatory markers in obese adults.

**Methods:**

Thirty-seven obese individuals aged 25 to 50 years were randomized to the hypocaloric diet exercise group or hypocaloric diet group. The participants underwent a 4-month follow-up. Electrical bioimpedance was used for body composition analysis. Biochemical data were analyzed by dry chemistry and insulin levels by ELISA. *ASC* gene expression from peripheral blood was performed using real-time PCR. Dietary data was collected through questionnaires and analyzed using the Nutritionist Pro™ software. Quantification of cytokines was conducted using Bio-Plex Pro™ Human cytokine. The Astrand-Ryhming test was used to estimate the maximum oxygen volume and design the moderate-intensity structured exercise program ~ 75% heart rate (HR)

**Results:**

After the intervention, both study groups significantly improved body composition (decreased weight, fat mass, waist circumference and abdominal obesity, *p* < 0.05). Besides, the diet-exercise group significantly decreased *ASC* mRNA expression, MCP-1, and MIP-1β inflammatory cytokines compared to the diet group (*p* < 0.05). While in the diet group, MCP-1 and IL-8 exhibited significantly decreased levels (*p* < 0.05). In the diet-exercise group, a positive correlation between the atherogenic index and waist circumference was found (*r* = 0.822, *p* = 0.011), and a negative correlation was observed between the delta of *ASC* mRNA expression and IL-10 levels at the end of the intervention (*r* = − 0.627, *p* = 0.019).

**Conclusion:**

Low-grade chronic inflammation was attenuated through individualized exercise prescription and our findings highlight the role of the *ASC* gene in the inflammation of obese adults.

**Trial registration:**

ClinicalTrials.gov, number NCT04315376. Registered 20 March 2020—retrospectively registered

## Background

Obesity is a global public health concern, representing at least 2.8 million deaths every year [1]. The major complications of obesity include chronic diseases like type 2 diabetes (T2D), cardiovascular (CVD), metabolic alterations, hypertension (HT), nonalcoholic steatohepatitis (NASH), and some types of cancer [2]. Genetic and environmental factors related to lifestyle have been described to increase obesity risk [3] and the secretion of circulating proinflammatory cytokines. Thus, obesity is associated with different degrees of low-grade chronic inflammation, also known as metabolic inflammation [4]. The development of obesity is related to the accumulation of large amounts of fatty acids in adipose cells, which leads to hyperplasia and hypertrophy, and thus, to the growth of adipose tissue. In response to these modifications, adipocytes move away from tissues with numerous blood vessels, resulting in hypoxia and the subsequent necrosis of adipose tissue [5]. Therefore, the damaged adipose tissue increases the infiltration of immune cells, the deficiency of immune activity, the increase of reactive oxygen species (ROS), and the amplified production of damage-associated molecular patterns (DAMPs) [6, 7].

The excess of proinflammatory cytokines has been linked with age-related diseases, such as heart failure, metabolic syndrome, insulin resistance (IR), T2D, HT, and asthma [7–9]. Particularly, IL-1β and IL-18 proinflammatory cytokines mature through the formation and activation of a protein complex, known as NLRP3 inflammasome. The release of PAMPs (patterns associated with pathogens) and DAMPs can activate the NLRP3 inflammasome, and once activated, it recruits a NOD (nucleotide-binding oligomerization domain)-like receptor, an apoptosis-associated speck-like protein containing a caspase recruitment domain (ASC), and caspase-1 [10]. The role of ASC protein is to provide stability to the NLRP3 inflammasome. Nonetheless, this protein has been reported as not having inflammatory activities outside of the protein complex. Thereby, it is suggested that the formation and activation of the NLRP3 inflammasome is limited to ASC regulation and it is expressed in monocytes, dendritic cells, neutrophils, and lymphocytes [10, 11]. This complex plays an important role in innate immunity and it responds to a great variety of microbial and endogenous products associated with stress and cellular damage; thus, it is considered a pivotal characteristic of inflammation [12]. Aside from its active participation in the local and systemic immune response, the inflammasome activates the production of the IL-1 family cytokines, which have an important role in the pathophysiology of various inflammatory diseases, such as atherosclerosis, T2D, rheumatoid arthritis, and some types of cancer [13, 14].

It is well known that physical exercise has many health benefits and it has different molecular responses depending on the frequency, intensity, and type of exercise [15]. Nevertheless, to our knowledge, there is no evidence regarding *ASC* gene activity in obese adults in response to lifestyle modifications. Therefore, the aim of this study was to evaluate the effect of a hypocaloric diet and a moderate-intensity structured exercise intervention on *ASC* gene expression and inflammatory markers in obese adults.

## Results

### Baseline characteristics

**Fig. 1:**
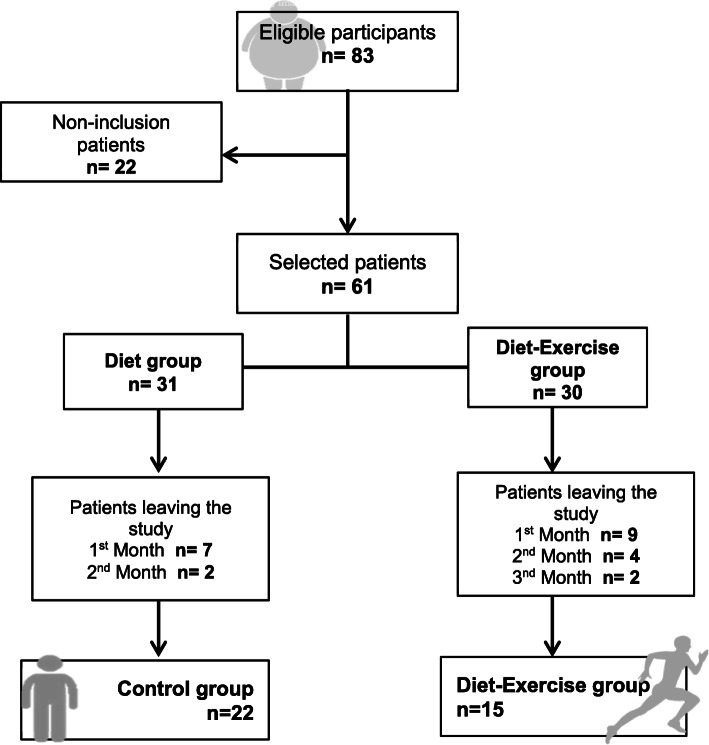
Flowchart of patient selection

A total of 61 participants were randomly divided into two groups: the diet group or the diet-exercise group. However, only 22 participants from the diet group and 15 participants from the diet-exercise group completed all the study. The main reason for the high dropout rate of participants was due to their lack of time to attend the training/nutrition appointment (Fig. 1). Furthermore, age was different between groups; therefore, subsequent statistical analyses comparing differences between study groups were adjusted for age. The baseline anthropometric and biochemical characteristics are shown in Table 1.

**Table 1:** Baseline anthropometric and biochemical characteristics

Variables	Diet group (***n*** = 22)	Diet-exercise group (***n*** = 15)	***p***
Age (years)	40 ± 8.1	33.6 ± 9.6	**0.032**
Sex (M/W)	6/16	5/10	1.000
Weight (kg)	92.4 ± 13.2	89.0 ± 10.5	0.389
MSM (kg)	29.6 ± 7.1	28.3 ± 6.8	0.580
FFM (kg)	53.3 ± 11.6	50.8 ± 11.2	0.510
BFM (kg)	39.1 ± 8.3	37.9 ± 8.5	0.681
BFP (%)	42.5 ± 7.5	42.9 ± 8.9	0.882
BMI (kg/m^2^)	33.6 ± 3.2	33.1 ± 2.8	0.604
WC (cm)	102.1 ± 10.1	96.4 ± 9.4	0.081
Abdominal fat (kg)	19.6 ± 3.2	19.4 ± 3.5	0.869
Glucose (mg/dL)	94.5 ± 14.6	91.1 ± 7.9	0.406
Total cholesterol (mg/dL)	187.3 ± 34.6	184.6 ± 30.4	0.800
Triglycerides (mg/dL)	167.4 ± 91.2	138.5 ± 81.8	0.313
HDL-c (mg/dL)	38.3 ± 7.8	43.4 ± 8.1	0.060
LDL-c (mg/dL)	115.1 ± 22.7	111.4 ± 27.4	0.642
VLDL-c (mg/dL)	33.5 ± 18.2	27.7 ± 16.4	0.309
Insulin (IU/dL)	23.0 ± 13.5	17.5 ± 8.5	0.152
HOMA-IR	5.5 ± 3.5	3.9 ± 2.11	0.123
Atherogenic index	5.09 ± 1.4	4.3 ± 0.8	0.070

The results are shown in mean ± standard deviation

*M* man, *W* woman, *MSM* musculoskeletal mass, *FFM* fat-free mass, *BFM* body fat mass, *BFP* body fat percentage, *BMI* body mass index, *WC* waist circumference, *HDL-c* high-density lipoproteins, *LDL-c* low-density lipoproteins, *VLDL-c* very low-density lipoproteins, *HOMA-IR* homeostatic model assessment—insulin resistance index

### Nutritional intervention

The baseline nutritional analysis did not show differences between groups. However, after the intervention with a hypocaloric diet (20% energy reduction), total energy, total sugar, total lipids, saturated fats, dietary cholesterol, trans fats, and sodium were decreased, and fiber, polyunsaturated fatty acids (PUFA), and the consumption of several vitamins were increased. Data are shown in Table 2.

**Table 2:** Nutritional evaluation in the study population

Nutritional variables	All population (***n*** = 37)	***p***
Kilocalories (kcal)	− 412.7 ± 739.8	0.133
Protein (gr)	− 11.4 ± 29.7	0.280
Protein (%)	1.6 ± 5.1	0.355
CH (gr)	− 53.8 ± 86.5	0.100
CH (%)	− 2 ± 10.9	0.595
Total lipids (gr)	− 14.3 ± 44.9	0.366
Total lipids (%)	1.8 ± 10.6	0.622
Total sugar (gr)	− 29 ± 49.4	0.116
Total dietary fiber (gr)	5.15 ± 8.1	0.092
Cholesterol (mg)	− 13.7 ± 144.4	0.782
Saturated fats (gr)	− 8.4 ± 18.5	0.208
Saturated fats (%)	− 0.9 ± 5.6	0.622
MUFA (gr)	− 6.1 ± 17.7	0.331
MUFA (%)	0.12 ± 4.9	0.941
PUFA (gr)	3.3 ± 11.7	0.419
PUFA (%)	3.6 ± 5	0.062
C vitamin (mg)	73.8 ± 120	0.102
D vitamin (mcg)	0.37 ± 3.6	0.767
E vitamin (mg)	0.25 ± 0.5	0.265
Sodium (mg)	− 354.8 ± 758.3	0.198

*CH* carbohydrates, *MUFA* monounsaturated fats acids, *PUFA* polyunsaturated fats acids

### Astrand-Ryhming test (A-R test)

All physical tests were performed without complications. After the exercise intervention program, no changes in maximum oxygen volume (VO_2max_) and heart rate were observed. However, the watts (W) employed during the test increased significantly. These findings represent a positive adaptation to a progressive three-phase moderate-intensity exercise program during 4 months conforming to the following periodization plan: frequency of 3 to 5 days, session time of 45 to 60 min, and intensity between ~ 65 and 75% HR, according to the training phase, considering that the significant increase in workload (W) does not represent an increased heart rate on A-R test (Table 3).

**Table 3:** Astrand-Ryhming test

Variables	Baseline (***n*** = 15)	Final (***n*** = 15)	***p***
HR (bpm)	133.6 ± 16.4	128.7 ± 21.6	0.346
VO_2max_ (ml/kg/min)	28.7 ± 7	31.5 ± 8.8	0.530
VO_2max_ (L)	2.6 ± 0.7	2.6 ± 0.85	0.906
Watt (W)	63.4 ± 15.5	76.5 ± 25.2	**0.048**

*HR* heart rate, *bpm* beats per minute, *ml/kg/min* milliliters/kilogram/minute, *L* liter, *W* watt

### Anthropometric and biochemical characteristics after the intervention

After 4 months of the intervention period, both study groups significantly improved their body composition; these data are shown in Table 4. The biochemical variables did not show significant changes after the intervention, but we observed a discrete tendency in atherogenic index (− 0.3 ± 0.5 *p* = 0.051) only in the diet-exercise group. Then, we compared the body changes between groups after the intervention and we observed a significant decrease in musculoskeletal mass (MSM) (*p* = 0.013) and fat-free mass (FFM) (*p* = 0.006) in the diet group compared to the diet-exercise group. Also, we observed a significant decrease in body fat percentage (BFP) (*p* = 0.029) and atherogenic index (*p* = 0.047) in the diet-exercise group compared to the diet group. In addition, out of the individuals who followed up the diet-exercise program, 26.7% of the subjects decreased abdominal obesity (according to the cutoff point of the International Diabetes Federation) and this change was statistically significant compared to the diet group (*p* = 0.026).

**Table 4:** Anthropometric changes after the intervention

Variables	Diet group (***n*** = 22) Δ	***p***	Diet-exercise group (***n*** = 15) Δ	***p***
Weight (kg)	− 3.9 ± 3.1	**0.000**	− 4.3 ± 4	**0.001**
MSM (kg)	− 1.13 ± 0.92	**0.000**	− 0.14 ± 0.23	0.555
FFM (kg)	− 1.94 ± 1.38	**0.000**	− 0.01 ± 0.46	0.978
BFM (kg)	− 1.95 ± 3.1	**0.010**	− 4.05 ± 3.7	**0.001**
BFP (%)	− 0.38 ± 2.3	0.460	− 2.8 ± 3.1	**0.004**
BMI (kg/m^2^)	− 1.3 ± 1.02	**0.000**	− 1.5 ± 1.4	**0.001**
WC (cm)	− 6.1 ± 3.1	**0.000**	− 6.2 ± 4.6	**0.000**
Abdominal fat (kg)	− 0.85 ± 1.5	**0.023**	− 1.9 ± 2.0	**0.002**

The results are shown in mean ± standard deviation

*MSM* musculoskeletal mass, *FFM* fat-free mass, *BFM* body fat mass, *BFP* body fat percentage, *BMI* body mass index, *WC* waist circumference, *Δ* = post data–pre data

### ASC mRNA expression

**Fig. 2:**
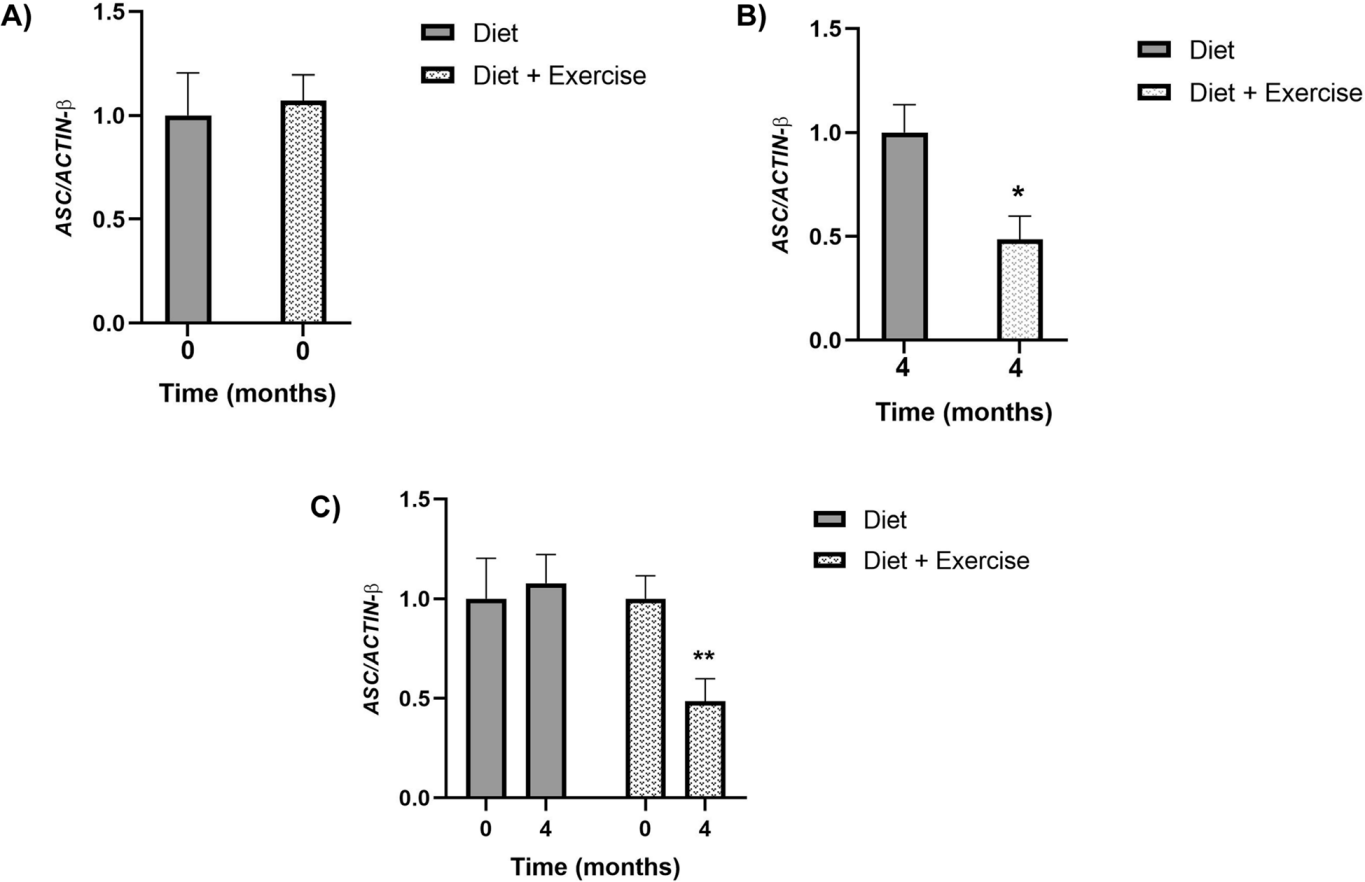
*ASC* mRNA expression by group over time. The results are shown as mean ± standard error of the mean. **a** *ASC* mRNA expression at baseline (*time 0)* between groups. **b** *ASC* mRNA at fourth month of intervention (*time 4*) between groups. **c** Change post-pre of each study group. *p = 0.043, **p = 0.030

We evaluated the *ASC* mRNA expression level in leukocytes at baseline time (*time 0*) of the intervention, and we demonstrated no significant differences between the study groups (1 ± 0.204 vs 1.07 ± 0.124, *p* = 0.990) (Fig. 2a). Subsequently, we evaluated the expression levels at *time 4* (4 months post-intervention), and we observed that the diet-exercise group significantly decreased *ASC* mRNA expression compared to the diet group (0.485 ± 0.112 vs 1 ± 0.134, *p* = 0.043) (Fig. 2b). Finally, the comparative analysis showed that the diet-exercise group significantly decreased *ASC* mRNA expression compared to baseline time (*time 0*) (1 ± 0.116 *vs* 0.532 ± 0.112, *p* = 0.030); however, these results were not observed in the diet group (1 ± 0.204 vs 1.07 ± 0.145, *p* = 0.812) (Fig. 2c). The analysis between groups was adjusted for age, gender, and baseline or final body fat percentage according to analysis.

### Cytokine measurements

**Fig. 3:**
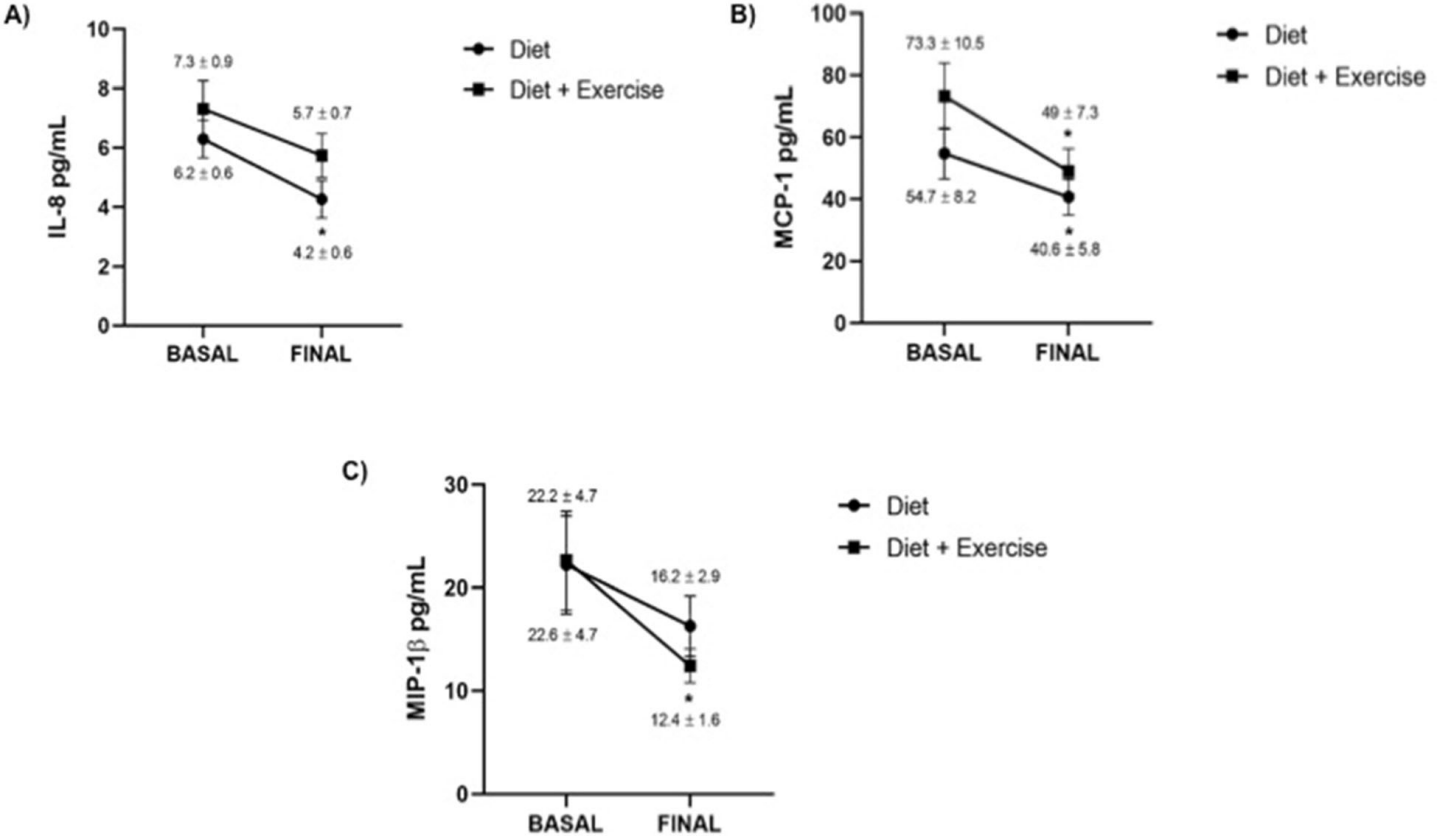
Post-intervention cytokine levels. The results are shown as mean ± standard error mean. **a** The diet group significantly decreased IL-8 levels after 4 months of diet intervention (*time 4*) (*p = 0.005). **b** Both groups significantly decreased MCP-1 levels after 4 months of the intervention (control group *p = 0.021, exercise group *p = 0.011). **c** Only the diet-exercise group significantly decreased MIP-1β after 4 months of the intervention (*p = 0.008)

After the intervention, the diet group significantly decreased IL-8 (6.2 ± 0.6 vs 4.2 ± 0.6 pg/mL, *p* = 0.005) (Fig. 3a) and MCP-1 (54.7 ± 8.2 vs 40.6 ± 5.8 pg/mL, *p* = 0.021) levels (Fig. 3b). Besides, the subjects who completed the diet-exercise program also decreased MCP-1 (73.3 ± 10.5 vs 49 ± 7.3 pg/mL, *p* = 0.011) (Fig. 3b) and MIP-1β levels (22.6 ± 4.7 vs 12.4 ± 1.6 pg/mL, *p* = 0.008) (Fig. 3c). Also, the IL1β cytokine levels in the diet group (1.2 ± 0.6 vs 0.8 ± 0.6 pg/mL) and diet-exercise groups (0.09 ± 0.08 vs 0.22 ± 0.12 pg/mL) as well as the IL-18 cytokine levels in the diet group (157.5 ± 75.2 vs 129.7 ± 40.4 pg/mL) and diet-exercise groups (95.4 ± 28.1 vs 95 ± 27) did not show significant differences in our population.

**Fig. 4:**
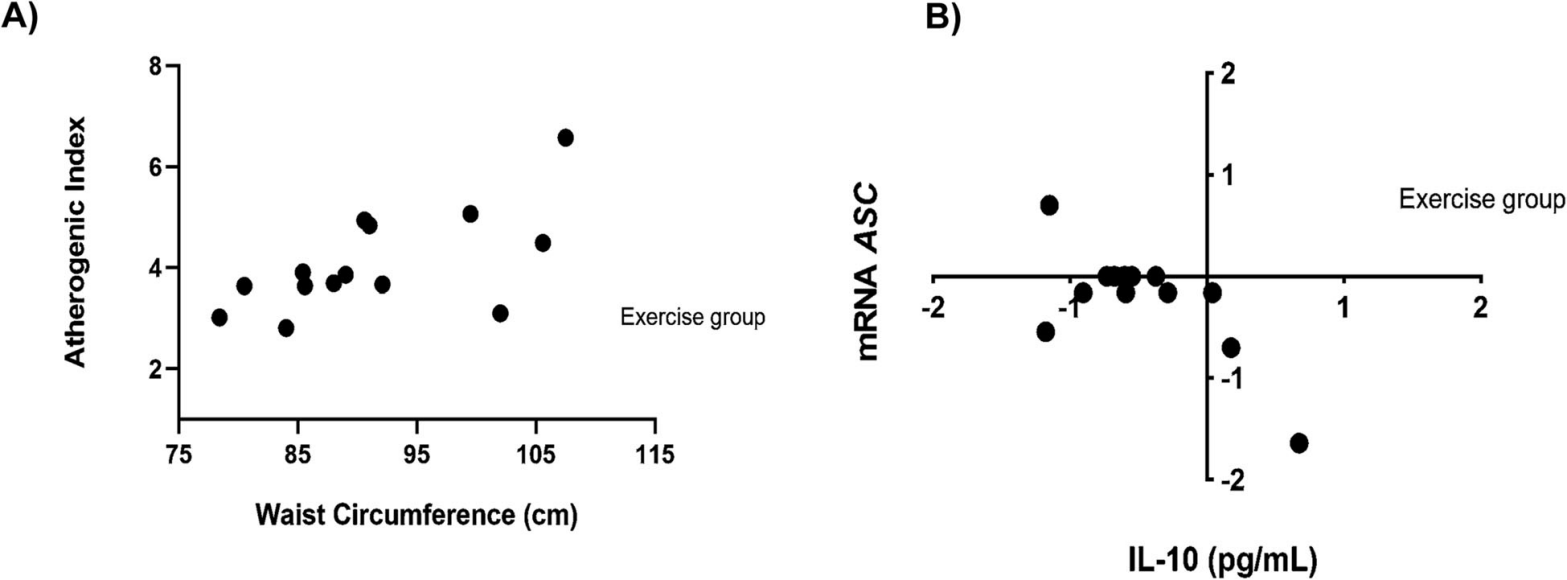
Correlation coefficients within the exercise-diet group. **a** The exercise-diet group showed a positive correlation between the atherogenic index and waist circumference (*p* = 0.011). **b** The same intervention group showed a negative correlation between the delta of *ASC* mRNA expression and the delta of IL-10 levels after the intervention (*p* = 0.035). Statistical analysis was adjusted for age, gender, and final fat percentage

The correlation analysis between the variables showed a positive correlation between the atherogenic index and waist circumference within the diet-exercise group (*r* = 0.822, *p* = 0.011) (Fig. 4a). Moreover, in the same study group, a negative correlation was observed between the delta of *ASC* mRNA expression and IL-10 levels at the end of the intervention (*r* = − 0.627, *p* = 0.019) (Fig. 4b). Additionally, the diet-exercise group showed a positive correlation between MIP-1β with abdominal obesity (*r* = 0.642, *p* = 0.033), BFM (*r* = 0.601, *p* = 0.050), BMI (0.657, *p* = 0.028), and BFP (*r* = 0.615, *p* = 0.044), and also between MCP-1 levels with abdominal obesity (*r* = 0.642, *p* = 0.033) and BFP (*r* = 0.769, *p* = 0.015). These correlations were not found in the diet group and all analyses were adjusted for age, gender and final body fat percentage.

## Discussion

Subjects with obesity go through a process known as low-grade chronic inflammation; in turn, this represents a risk factor for the development of metabolic disorders and several diseases, such as non-alcoholic steatohepatitis (NASH) and even cancer. There are different nutritional strategies for the obesity approach, among which lifestyle changes are the most successful [16]. However, it is important to note that regular exercise has an important anti-inflammatory response. Therefore, we evaluated the effect of a 4-month hypocaloric diet and exercise program on *ASC* mRNA expression and inflammatory markers in obese adults, and we demonstrated that *ASC* mRNA expression was decreased in obese participants after the diet-exercise program, and a significant difference was found compared to the diet group at the end of the intervention. These results show that exercise has an effect on the expression of the *ASC* gene.

Other studies have demonstrated hypermethylation of the *ASC* gene in heart failure patients and in older individuals after exercise, and in turn, methylation was related to decreased expression of *ASC* and *IL-1* family. Therefore, exercise may play an important role in the epigenetic modification of the *ASC* gene [8, 9, 17, 18]. Although in this study we did not measure IL-1β or IL-18 expression, we quantified both cytokines; however, no significant differences were found probably because our study population did not have another metabolic disease.

Thus, we propose the *ASC* gene as a molecular marker in response to the exercise intervention to attenuate inflammation in obese individuals and prevent the consequences of low-grade inflammation, since we also observed a negative correlation between the delta of *ASC* mRNA expression and IL-10 levels in the diet-exercise group.

Interleukin-10 (IL-10) is an important anti-inflammatory cytokine that is associated with the immune response [19], the inhibition of IL-1α, IL-1β, IL-6, IL-8, TNF-α proinflammatory cytokines [20], and the suppression of the NFk-B signaling pathway [21]. Previous studies have reported that exercise training is associated with a higher IL-10 cytokine production [22, 23] stimulated by higher circulating Treg cell levels and an anti-inflammatory state [24]. Although there is not enough evidence, Treg cells could have a possible mechanism to regulation of *ASC* expression through exercise training and IL-10 cytokine participation [25]. Since this study found a correlation between the deltas of IL-10 and ASC, in this context, ASC is suggested as the possible regulatory molecule between exercise, Treg cells, obesity, and inflammation. However, more research is necessary to support this.

In addition, the hypermethylation of the *ASC* gene has been correlated with better results of the 6-min walk test [17]. This seems to indicate that cardiorespiratory fitness (CRF) could be related with *ASC* regulation. Also, other studies in overweight or obese participants have reported that CRF has an inverse association with inflammation mediators in peripheral blood lymphocytes, monocytes, neutrophils, and non-classical monocytes [26–28]. However, given the significance of low-grade chronic inflammation in the pathogenesis of various diseases, the association between fitness and inflammation in obese patients is still unclear.

Furthermore, we also found MCP-1 and MIP-1β decreased levels after the exercise program, and these cytokines are synthesized by monocytes and macrophages [29, 30], the main cells where NLRP3 inflammasome is activated [10]. Both cytokines are related to cardiovascular alterations and atherogenic development and are stimulated by proinflammatory cytokines, such as IL-1β. Therefore, we could expect from our findings that the exercise program had a role in the decrease of NLRP3 inflammasome activation through *ASC* downregulation, MCP-1, and MIP-1β; thus, improving the anti-inflammatory profile through the IL-10 cytokine.

Moreover, visceral adipose tissue surrounding the internal organs has inflammatory activity since it is associated with a greater number of proinflammatory cells in the tissue [31]. Therefore, the decreased levels of MCP-1, MIP-1β, and IL-8 cytokines in our study also may be in part due to the abdominal fat loss, since these results showed a positive correlation between them (*p* < 0.05).

In this study, both intervention groups improved body composition, which was consistent with other studies where the effect of hypocaloric diets accompanied or not by 3 months of an exercise intervention program decreased the same variable [32, 33]. These results are expected and partly explained due to the energy restriction involving the activation of lipolytic metabolic pathways, which increase the use of adipose tissue as stored energy [34]. Besides, if the demand for energy increases, as in exercise, the mobilization of fatty acids from stored adipose tissue also increases [35].

In addition, we observed that the participants who performed the diet-exercise program did not have significant changes in musculoskeletal and fat-free mass compared to the diet group, so this indicates that the weight loss was mainly in fat mass. This finding demonstrates some of the additional benefits of exercise in weight loss management since adding exercise training to energy restriction results in favorable body composition changes in obese subjects. Our results are in accordance with other authors who reported that diet-exercise interventions for 6 months preserved lean body mass or skeletal muscle mass compared to only diet interventions in obese adults [36, 37].

Abdominal obesity has been associated with systemic inflammation [38] and metabolic risk [39]. However, in our study, we found that 26.7% of the participants who performed exercise decreased abdominal obesity, and this was significant when compared to the diet group. Our result is consistent with other studies, which show that regular exercise reduces visceral fat, independent of weight reduction [40, 41]. Moreover, O’Donovan et al. found that overweight subjects with better physical fitness had lower visceral fat than the overweight unfit group [42]. Our results and the evidence mentioned above highlighted the effect of exercise on abdominal fat loss, suggesting that systemic inflammation decreased regardless of weight loss.

In our study, the diet-exercise group showed a tendency to decrease the atherogenic index at the end of the intervention, and a statistical difference was found when we compared the atherogenic index change between groups. Our finding is similar to other results that show an association of exercise with the atherogenic index of plasma after 12 weeks of aerobic exercise in obese individuals [43] as well as a lower atherogenic index in subjects who performed regular exercise compared to sedentary subjects [44]. Lastly, an inverse association was found between the atherogenic index and physical activity levels [45].

In addition, there is evidence that associates obesity as a precursor of atherosclerosis, particularly central adiposity, which is usually determined by waist circumference [46]. In this sense, we found a positive correlation between the atherogenic index and waist circumference in the diet-exercise group, and it is important to mention that this group had a greater abdominal fat loss. Therefore, our results support the evidence that the loss of abdominal obesity could decrease the risk of atherosclerosis and, subsequently, the risk of cardiovascular diseases. This finding highlights the benefits of exercise as a cardio-protector factor.

Given our findings, we believe it is important to consider the measurement of ASC protein, *ASC* methylation, and the expression of the IL-1 family in obese subjects. Also, there were limitations in our study such as the small sample size and the high dropout rate in the exercise group, which could be due to the lack of commitment and poor adherence to achieve a healthy habit over a long period of time, as well as the subjective method used to quantify the VO2_max_, and accelerometers were not used in each exercise sessions. Future researches are needed to be done in the same population to support our results and the mechanism of *ASC* downregulation through exercise and including more markers related to low-grade chronic inflammation pathway.

## Conclusion

Our study demonstrated that the structured moderate-intensity exercise program attenuates low-grade chronic inflammation and highlights the role of the *ASC* gene in the inflammation of obese adults. This study emphasizes the importance of exercise for weight management in individuals with obesity to protect from cardiovascular disease, type 2 diabetes, and metabolic syndrome. Finally, a practical application of *ASC* as a molecular marker in peripheral blood of obese subjects could be used to assess low-grade chronic inflammation due to its response to lifestyle modifications.

## Methods

### Subjects

A randomized clinical trial (ClinicalTrials.gov, Number NCT04315376) was conducted at the Institute of Translational Nutrigenetics and Nutrigenomics of the University of Guadalajara. The study was performed from February 2018 to February 2019. Eighty-three participants were recruited through flyers and social media invitations; however, 61 met the inclusion criteria (18 male and 43 female). The inclusion criteria were mestizo participants from West Mexico, age between 25 to 50 years with obesity (BMI ≥ 30 kg/m^2^), with a waist circumference greater than 80 cm in women and 90 cm in men, a sedentary lifestyle according to the World Health Organization (WHO) [47], and subjects without history of medication for at least 1 year. Non-inclusion criteria were pregnant or breastfeeding women, diagnosis of diabetes, cardiovascular disease, and cancer, tobacco and alcohol (consumption ≥ 40 g of alcohol per day for men and ≥ 20 g for women) consumption, and muscle or joint injury. All the participants signed a written informed consent. This study was approved by the Ethics and Biosafety Committee of the Health Sciences Center, University of Guadalajara (Registration number CI-08518), and was carried out according to the Declaration of Helsinki (2013).

### Intervention

The intervention consisted of a 4-month follow-up period. The participants were randomly assigned to the diet and exercise program intervention (diet-exercise group) or only diet program intervention (diet group). Also, we defined the baseline measures as time 0 and the final measures (4th month) as time 4.

### Astrand-Ryhming test

The participants who performed the diet-exercise intervention completed an Astrand-Ryhming submaximal test described by Astrand [48] on RECK MOTOmed viva2 cycle ergometer to estimate the VO_2max_ before the exercise intervention program (50–75 W for woman and 50–100 W for man) and to design the moderate-intensity structured exercise program based on the heart rate, which was monitored during the test in each individual. Once the individuals completed the training program, the final Astrand-Ryhming test was performed considering the individuals as trained; therefore, the measured watts were greater according to the Astrand protocol (75–100 W for women and 100–150 W for men).

### Exercise program intervention

A personal trainer certified by the American College of Sports Medicine designed a progressive three-phase moderate-intensity exercise program: (1) conditioning phase: 45 min/day, 3 days per week for 5 weeks (~ 65%HR); (2) progression phase: 1 h/day, 4 days a week for 8 weeks (~ 70–75%HR); and (3) maintenance phase: 1 h/day, 5 days per week for 3 weeks (~ 75%HR). The main exercises consisted of improving aerobic (walking and jogging), speed (functional exercise circuits and short races), and resistance performance using dumbbells (weighing less than 5 kg). During all sessions, breathlessness and fatigue were measured with the Borg CR10 grade scale (0–10). The personal trainer supervised three sessions per week within the sports facilities of the University Center to ensure that all exercises were performed with an appropriate technique. For the two unsupervised training days, participants were given the detailed training program to perform in the house or park.

### Nutritional intervention

The nutrition service carried out the dietary intervention and follow-up evaluation each month. The validated 24-h diet record and the 3-day dietary food record questionnaire (2 days during the week and 1 day of the weekend) were used to collect the dietary information. Participants were instructed to provide the correct description of their habitual food intake. The nutritionist showed food scale models from Nasco® to enhance the accuracy of the portion sizes based on Mexican food composition tables. The Nutritionist Pro™ software (Axxya Systems, Woodinville, WA) was used to estimate the energy intake and food consumption quantification by a trained nutritionist. The nutritional intervention consisted of a hypocaloric diet 20% total energy expenditure reduction estimated by the *Mifflin-St.Jeor* formula [49] using the current weight, with a nutrient distribution as follows: 50% carbohydrates, 30% lipids, and 20% proteins.

### Anthropometric and biochemical measurements

Height and weight were measured after an 8–12-h fast and with participants wearing light clothes. Height measurements were taken using a stadiometer (Rochester Clinical Research, NY, USA). Waist circumference was measured using a Lufkin Executive® tape and body composition by electrical bioimpedance (InBody 370, Biospace Co. Seoul, Korea) every month of the intervention period.

Peripheral blood samples were taken after an 8–12-h fast and immediately centrifuged at 3500 rpm to obtain serum. Biochemical variables were performed using a dry chemistry analyzer (Vitros 250 Analyzer, Ortho-Clinical Diagnostics, Johnson & Johnson Services, Inc., Rochester, NY, USA). Low-density lipoprotein cholesterol (LDL-c) was calculated using the Friedewald formula except when triglycerides levels were higher than 400 mg/dL. The atherogenic index was calculated using the formula [total cholesterol (mg/dL)/HDL-c (mg/dL)], and for cardiovascular risk, values > 3 in women and > 3.5 in men were considered. Serum insulin levels were determined using Insulin Model ELISA kit, Catalog: CT-600101A (International Diagnostics, S.A de C.V) following the supplier’s instructions. The homeostatic model assessment of insulin resistance (HOMA-IR) was calculated as described by Matthews [50]. A HOMA-IR value > 2.5 was considered as insulin resistance. The biochemical variables were measured every month during the intervention period.

### ASC mRNA expression and cytokine levels

The *ASC* mRNA expression from peripheral blood and cytokine levels from serum samples were measured before the start (time 0) and at the end of the intervention (time 4). Total RNA was extracted from 5 ml of peripheral blood samples, and the quantification and purity were measured by Nanodrop 2000 UV (Thermo Fisher Scientific, Waltham, MA). The cDNA synthesis was performed using 1 μg of total RNA according to standard techniques [51] of Invitrogen™, Carlsbad, CA.

Quantitative real-time PCR was performed using Hs00203118_m1 TaqMan® probes (Applied Biosystems, Foster City, CA) in a Light Cycler 96 system considering standard PCR conditions (Roche, Mannheim, Germany) to analyze *ASC* mRNA relative expression by 2^−ΔΔCt^ method. The amplification reactions were performed in duplicate using the *ACTβ* gene (Hs01060665_g1, Applied Biosystems, Foster City, CA) as a constitutive gene to normalize the samples.

The quantification of proinflammatory and anti-inflammatory cytokines was performed using Bio-Plex Pro™ Human cytokine Standard 17-Plex, Group I kit (Cat. 10014905, Bio-Rad-Laboratories, Hercules, CA) following the supplier’s instructions and read immediately using the MAGPIX™ analyzer. The IL-18 cytokine was measured with Interleukin-18 Human ELISA kit (Biovendor, Brno, Czech Republic) in accordance to the manufacturer’s instructions.

### Statistical analysis

The sample size was calculated using the mean difference formula for clinical trials in order to detect statistical differences in *ASC* gene expression in peripheral blood using the data from the Dang et al. study [52]. To achieve a statistical power of 80% and an alpha of 5%, a sample size of 13 participants in each study group was required. However, considering the predicted loss of participants during the study, more than 13 individuals were included per study group. Shapiro-Wilk test was used to evaluate data normality and the Levene’s test to verify the homogeneity of the variables. Quantitative variables are expressed as mean ± standard deviation (SD) or standard error of the mean (SEM) when the analysis was adjusted for co-variables. Statistical differences between groups were analyzed using the unpaired Student’s *t* test and paired Student’s *t* test or Wilcoxon test to evaluate changes between baseline and final time depending on the normality of the variables, and we used repeated measures ANOVA to observed differences over time. Pearson and Spearman correlation coefficients were considered normality dependent. A *p* value < 0.05 was considered statistically significant. All statistical analyses were performed using the software SPSS v.22.0 (IBM, Chicago, IL).

## Data Availability

All data generated or analyzed during this study are included in this published article.

## Abbreviations

VO_2max_: Maximum oxygen volume
T2D: Type 2 diabetes
CV: Cardiovascular
HT: Hypertension
IR: Insulin resistance
CRF: Cardiorespiratory fitness
NASH: Nonalcoholic steatohepatitis
DAMPs: Damage-associated molecular patterns
ASC: Apoptosis-associated speck-like protein containing a caspase recruitment domain;
NOD: Nucleotide-binding oligomerization domain-like receptor
MSM: Musculoskeletal mass
FFM: Fat-free mass
BFM: Body fat mass
BFP: Body fat percentage
BMI: Body mass index
WC: Waist circumference
HDL-c: High-density lipoproteins
LDL-c: Low-density lipoproteins
VLDL-c: Very low-density lipoproteins
HOMA-IR: Insulin resistance index
CH: Carbohydrates
MUFA: Monounsaturated fats acids
PUFA: Polyunsaturated fats acids
